# SARS-CoV-2 Variants and Clinical Outcomes of Special Populations: A Scoping Review of the Literature

**DOI:** 10.3390/v16081222

**Published:** 2024-07-30

**Authors:** Achilleas Livieratos, Charalambos Gogos, Karolina Akinosoglou

**Affiliations:** 1 Independent Researcher, 15238 Athens, Greece; 2 Department of Medicine, University of Patras, 26504 Rio, Greece; cgogos@med.upatras.gr (C.G.); akin@upatras.gr (K.A.); 3 Department of Internal Medicine and Infectious Diseases, University General Hospital of Patras, 26504 Rio, Greece

**Keywords:** SARS-CoV-2 variants, clinical outcomes, immunocompromised, HIV, pediatric, chronic liver disease

## Abstract

The ongoing COVID-19 pandemic has significantly impacted special populations, including immunocompromised individuals, people living with HIV (PLWHIV), pediatric patients, and those with chronic liver disease (CLD). This scoping review aims to map the clinical outcomes of these vulnerable groups when infected with various SARS-CoV-2 variants. The review identifies trends and patterns, noting that early variants, such as Alpha and Delta, are associated with more severe outcomes, including higher hospitalization and mortality rates. In contrast, the Omicron variant, despite its increased transmissibility, tends to cause milder clinical manifestations. The review highlights the necessity for ongoing surveillance and tailored healthcare interventions due to the heterogeneity of patient populations and the evolving nature of the virus. Continuous monitoring and adaptive healthcare strategies are essential to mitigate the impact of COVID-19 on these high-risk groups.

## 1. Introduction

COVID-19 continues to be a major concern for both individual health and broader healthcare systems [1]. Emerging mutations of the virus are highly transmissible and are capable of causing reinfections, which is a significant concern for healthcare professionals [1,2]. These variants are capable of causing severe illness and fatalities. In order to address this phenomenon, it is critical to prioritize the authorization of updated immunization protocols, as well as develop targeted drugs to combat evolving strains [1,2].

Although some countries have chosen to adopt a strategy of living with the virus as a preventive measure, special populations, including immunocompromised individuals, patients living with HIV (PLWHIV), and other vulnerable groups, still require increased surveillance due to their vulnerable state [1,2]. Therefore, the implementation of long-term measures such as social distancing, wearing masks, vaccination, and early diagnosis remain relevant for these special populations to some degree even to this day [1].

In the second half of 2020, two separate lineages emerged. [2]. These were subsequently identified as the Alpha variant and Beta variant [2]. The United Kingdom first reported the Alpha variant on 14 December, while South Africa announced the detection of the Beta variant on 18 December [2,3]. On 9 January 2021, Japan notified the WHO of the P.1 variant, which had been identified in travelers arriving from Brazil [2,3]. The P.1 lineage was later designated as Gamma [2,3]. Another variant, Delta, was initially observed in India, and by mid-2021, it had been detected in at least 132 countries and become the dominant strain causing the rapidly increasing number of reported cases worldwide [2,3]. Compared to the Alpha, Beta, and Gamma strains, the Delta variant demonstrated increases in transmissibility of approximately 55%, 60%, and 34%, respectively [2,3,4]. By the end of 2021, the Omicron strain had been reported in Africa [2,3,4]. For several months following the introduction of the Omicron strain, both the Delta and Omicron variants co-circulated, posing dual threats [2,3,4]. However, Omicron eventually became the dominant variant in numerous countries, displacing Delta by early 2022 [2,3,4]. The Omicron strain had several spike protein mutations which increased its transmissibility compared to previous variants [4]. Since 2023, numerous subvariants have emerged such as XBB.1.5 and XBF, which continue to be monitored [2].

Regardless of the strain involved, the implications of this viral infection have been extensively explored in various studies [5,6,7,8,9,10,11,12]. Kartsonaki et al. analyzed SARS-CoV-2 virus-positive samples from 679,194 patients from 52 countries and concluded that approximately 15.9% required admission to the intensive care unit (ICU), with around one-third of these admissions occurring on the same day of hospitalization [5]. The overall case–fatality ratio varied across countries and time, but the study also demonstrated that males had a higher likelihood of mortality compared to females [2,5]. Moreover, age was significantly associated with an elevated risk of death [2,5,6]. Apart from asthma and rheumatologic disorders, all other reported comorbidities were linked to an increased risk of mortality [2,5,6,7].

Ongoing research is investigating the impact of SARS-CoV-2 variants across various metrics, including severity, hospitalization, death, and ICU admission [5,6,7,8,9,10,11,12]. Notably, the Alpha and Beta variants have demonstrated a significant probability of causing severe clinical outcomes versus non variants of concern [5,6,7,8,9,10,11,12]. Research has further indicated that the Delta variant is linked with higher rates of ICU admissions and poor outcomes [5,6,7,8,9,10,11,12]. Conversely, evidence suggests that the Omicron variant exhibits a milder pathogenicity, resulting in reduced disease severity, but an increased transmissibility compared to previous strains [5,6,7,8,9,10,11,12].

As SARS-CoV-2 continues to evolve, along with its impact on various special populations, it is crucial to accurately document these developments at the clinical level. Investigating clinical outcomes is particularly important when studying special populations such as immunocompromised individuals, PLWHIV, children, and patients with chronic liver disease (CLD) [13]. In this work, the repercussions of distinct virus strains on clinical outcomes within specific special populations are explored [13].

## 2. Materials and Methods

To understand the correlation between infective variant and patient health status, we investigated several parameters including population cohort, variant of concern, and hospitalization/mortality outcomes [13,14,15,16]. Our research question aimed to investigate how different variants of the SARS-CoV-2 virus are associated with specific clinical outcomes in infected individuals across four different special populations. These special populations included immunocompromised, PLWHIV, pediatric patients, and CLD patients. A comprehensive literature search on the PubMed database was conducted, including articles published from January 2020 to May 2024. Initially, various search algorithms were tested, including terms such as variants, mutations, outcomes, alongside each special population cohort (OMICRON OR DELTA OR GAMMA OR ALPHA VARIANT AND MORTALITY OR HOSPITALIZATION AND IMMUNOCOMPROMISED OR HIV OR CHRONIC LIVIER DISEASE OR PAEDIATRIC) we intended to examine. However, following the unavailability of relevant manuscripts using such an approach, we focused our search on the following search strings as follows: (SARS-CoV-2 VARIANTS AND IMMUNOCOPROMISED AND OUTCOMES; SARS-CoV-2 VARIANTS AND HIV AND OUTCOMES; SARS-CoV-2 VARIANTS AND CHRONIC LIVER DISEASE AND OUTCOMES; SARS-CoV-2 VARIANTS AND PEDIATRIC AND OUTCOMES). This approach provided focused findings for each special population investigated. The rationale for selecting these studies was either large population size, or a variety of SARS-CoV-2 variants investigated, or patients with chronic diseases of varying degrees of severity. Subsequently, two independent investigators screened, selected, and analyzed the database output and excluded obsolete articles, omitting them from consideration. A third researcher was involved to resolve any potential disagreements. We included articles focusing on variants and special populations in relation to clinical outcomes and excluded essays, opinions, editorials, and case reports.

**Figure 1:**
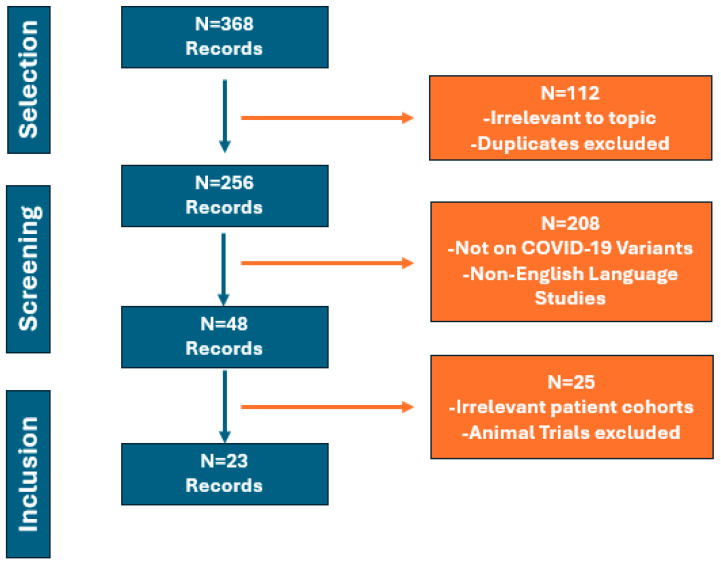
Articles inclusion flowchart.

Initially, the titles and abstracts of 368 articles were assessed. After a comprehensive search and filtering process, we subsequently excluded 112 articles based on their titles and abstracts as they were unrelated to the topic of interest as well as duplicates. Subsequently, another 208 articles were excluded as they did not focus on the clinical outcomes of different SARS-CoV-2 variants. Any non-English manuscripts were also excluded. Forty-eight records were ultimately reviewed as full manuscripts to examine their eligibility for inclusion. Twenty-five articles were excluded in this final step as they focused on animal studies, or the patient cohorts included were not directly applicable for the purposes of this work. Consequently, twenty-three studies were considered relevant for the purposes of this scoping review. A schematic of the literature investigation and selection is presented in Figure 1.

Most studies included (n = 10) were conducted in the United States of America, followed by the United Kingdom (n = 4). Regarding studies that included special populations, 9 studies were on immunocompromised patients, 6 on PLWHIV, 4 on pediatric patients, and 4 on CLD patients.

## 3. Discussion

Upon conducting an independent analysis of clinical outcomes related to different variants in special populations, we identified that patients with similar demographic and comorbidity profiles were mostly impacted by specific variants (Alpha/Delta) [13,14,15,16,17,18]. A recent large multinational trial aimed to determine the rates of mortality and ICU admissions [13,19]. This study reported that patients exposed to the Alpha strain are more likely to be hospitalized and have a fatal outcome [19]. Similarly, Veneti et al. analyzed 23,169 cases and discovered poor outcomes associated with infections of the Alpha and Beta strains [6,13].

Another important study discovered that the Delta variant was associated with higher oxygen requirements and even higher ICU admissions and mortality rates compared to the Alpha and Beta variants. Additionally, this variant exhibited an increased viral load and prolonged viral shedding [20]. However, conflicting reports indicated that Delta did not result in more cases of hospitalizations, ICU admissions, or deaths among adults [6,8,13]. Those conflicting findings were partly attributed to unvaccinated individuals, including adults below the age of 50 [13,20]. In contrast, individuals infected with the Gamma strain often fall within the age range of 45 to 64 years and are more likely to develop upper respiratory symptoms compared to patients infected by other variants [17,18]. However, despite not being as severe, the outcomes following an Omicron variant infection remain poor among certain special population groups, such as older adults, children, and immunocompromised individuals [21,22,23,24,25]. In pediatric cases infected by the Omicron variant, there is an increased likelihood of heightened immune responses and severe neurological disorders [26,27]. Additionally, the higher transmission rates of this variant continue to result in more pediatric patients becoming infected [26,27]. Due to the narrower upper airways, inflammation of the larynx often leads to significant clinical manifestations in younger children [26,27]. Following the dominance of the Omicron variant, there was a notable rise in hospitalization rates among children aged 0–4 [1,26,27].

### 3.1. Immunocompromised Patients

Due to weakened immune responses, immunocompromised individuals face a heightened risk of developing severe outcomes and complications from infectious diseases [28,29,30,31,32,33]. At the population level, all immunocompromised individuals face a higher likelihood of hospitalization and poor clinical outcomes associated with COVID-19 [34,35]. According to a case–control study (Delta and Omicron variants), immunocompromised adults were more than twice more likely to be hospitalized (81% hospitalized during the Omicron period) versus controls [36,37]. However, the severity of COVID-19 varies significantly among immunocompromised individuals [36,37]. Various factors, including the nature of their immune dysfunction, any medications suppressing immune function, and other co-morbidities, all contribute to the complex mechanisms that determine the susceptibility to severe virus infection [28].

To investigate the association between patients with immune system dysregulation and ICU admission further, data from adults over 18 years of age, currently hospitalized with a laboratory-confirmed infection, were obtained [35]. The study covered the period from 1 March 2020 to 28 February 2022 [35]. Additionally, the study examined possible connections between immunization status, ICU admission, and in-hospital mortality from 1 March 2021 to 28 February 2022 [35]. Among the hospitalized individuals included in this study during these two years, 12.2% were classified as being immunocompromised out of the 22,345 patients [35]. 11.1% were hospitalized during the pre-Delta period: 10.9% during the Delta period, and 17% during the Omicron period [35]. The analysis reported that unvaccinated, immunocompromised patients had higher adjusted odds ratios for ICU admission (aOR = 1.26; 95% CIs: [1.08–1.49]) and in-hospital mortality (aOR = 1.34; 95% CIs: [1.05–1.70]) compared to controls [35]. Moreover, for patients with specific immunocompromising conditions, in-hospital mortality (aOR) was twice as likely than for patients with AIDS [35]. Equally, individuals undergoing immunosuppressive therapy were more likely to experience poor clinical outcomes, especially patients diagnosed with multiple myeloma or those who had undergone a solid organ transplant [35]. Conversely, patients with an immunoglobulin deficiency did not report as many poor outcomes as other groups [35].

During the Delta period and prior to its emergence, it was observed that individuals with compromised immune systems generally faced a higher risk of mortality, irrespective of their vaccination status, compared to those who were not immunocompromised [35]. However, more recently, during the Omicron variant-dominant period, the mortality risk among individuals, whether they were immunocompromised or vaccinated, remained similar [35].

Recently, a retrospective investigation reported the impact of immune disorders and/or immunosuppressants on infection prognosis [38]. This study reported that having an immune disorder alone led to an odds ratio (OR) of 1.13 (95% CIs: [1.09–1.17]) higher likelihood (across the Ancestral, Alpha, Beta, Gamma, Delta, and Omicron variants) of developing life-threatening conditions compared to those without an immune disorder [38]. Among patients with preexisting immune disorders using immunosuppressants, the likelihood of developing life-threatening conditions increased even further to OR 1.35 (95% CIs: [1.29–1.40]) compared to controls [38]. Although criteria like clinically vulnerable status offer general guidance on frailty, it is vital to recognize the degree of heterogeneity present in patients that even share similar immunological disorders [28,39,40,41]. This is visible in PLWHIV, whereby the evidence for the risk of poor outcomes may be conflicting at times, particularly for specific sub-populations [28,41,42,43].

Another study that investigated the clinical outcomes of each major variant, and included special populations within its cohort, included a total of 2779 patients [44]. Each patient was classified into one of four categories: the Alpha variant (n = 1153), the Gamma variant (n = 122), the Delta variant (n = 808), or the Omicron variant (n = 696) [44]. The likelihood of hospitalization or mortality was not significantly different among patients infected with any of those variants, besides the Omicron variant [44]. In those patients, significantly lower rates of hospital admission, oxygen requirement, and ICU admission were reported compared to the other strains [44]. Interestingly, the hospitalization rate for breakthrough infections was found to be similar across different variants [44]. Furthermore, no significant differences in disease severity were observed between the subvariants belonging to the omicron strain [44].

According to a study by de Prost et al., patients with compromised immune systems have increased rates of mortality and complications compared to individuals with normal immune function in both the Omicron and Delta variants [45]. Delta-exposed individuals did require more EMCO support when compared to those with Omicron infections (18% vs. 6%) [45]. A comprehensive analysis of healthcare claims data in the United States spanning from April 2020 to March 2022 (all variants) reported that 23.5% of immunocompromised individuals required hospitalization [46,47]. During the Omicron period, although immunocompromised individuals accounted for 4% of the patient cohort in Esper et al.’s study, they were associated with 22% of all COVID-19 hospitalizations [44].

Moreover, among the Omicron strain infections, patients with compromised immune systems exhibited more frequent occurrences of organ failure and higher 28-day mortality rates [45,46]. Despite the potential benefits conferred by vaccines, immunocompromised patients still encounter challenges in terms of therapeutic effectiveness and post-hospitalization outcomes (Table 1) [1].

In conclusion, earlier variants contributed to an increased mortality in immunocompromised populations. Importantly, the heterogeneity of this patient population coupled with increased immunization rates later in the pandemic are relevant factors that substantially contribute to the clinically diverse outcomes reported by the included studies.

### 3.2. PLWHIV

Approximately 38.4 million individuals worldwide are presently infected with HIV, with about 75% of them making use of antiretroviral treatment (ART) [51]. The pandemic has significantly interrupted HIV management and treatment, especially in low-income nations [51]. During 2020 alone, treatment referrals were reduced by one-third, and there was an over 40% drop in testing, compared to the same period in 2019 [51]. Importantly, PLWHIV with a CD4 cell count above 200 are not considered immunocompromised [51,52].

To date, conflicting data exist regarding individuals co-infected with HIV and COVID-19. Varying results have been observed across different regions and within specific geographical territories [39,52,53,54]. Early studies conducted with small groups of patients during the Ancestral strain period suggested that PLWHIV had no greater risk of severe disease and mortality [39,52,53,54]. Research has nevertheless now reported a correlation between untreated PLWHIV and an increased mortality risk (pre-Omicron period) following SARS-CoV-2 infection, despite earlier studies reporting no significant association in patients on antiretroviral treatment [39,40,41,42,43]. When collective data from multiple centers in the UK were analyzed, it was discovered that PLWHIV, during the Ancestral strain period, had almost a three times greater probability of causing mortality (Hazard Ratio: 2.90; 95% CIs: [1.96–4.30]) compared to controls [55]. Notably, among PLWHIV, black ethnicity demonstrated a significantly greater risk versus other ethnic backgrounds [55].

Research from South Africa has now also reported a direct correlation between specific viral strains and poor outcomes among HIV infected patients [43,51]. There was no significant difference between PLWHIV and controls in terms of mortality rates during the Alpha/Beta-dominant period [43,51]. These death rates remained relatively stable throughout 2021, during which time pre-Omicron variants were dominant [43,51]. However, after 2021, during the circulation of Omicron, the difference in the mortality rate between PLWHIV and controls jumped by a factor of more than 2 (19.8% vs. 8%) [43,51]. The consistently elevated death rate among PLWHIV may be partly attributed to the reduced immunization at the time, when just a third of the South African population was administered both vaccine doses [43,51].

A study conducted during the pre-Delta period by the WHO Global Clinical Platform, focused on hospitalized COVID-19 patients [43,51]. The study demonstrated a 15% greater probability of poor clinical outcomes in PLWHIV and around a 40% greater probability of in-hospital mortality [43]. Individuals above the age 18, with co-morbidities such as malignancies or other chronic diseases, were identified as high-risk patients for mortality during hospitalization [43]. However, caution should be exercised when extrapolating findings from this and similar African studies to western countries, as the applicability of their findings may vary [43,51].

Research from New York during the Ancestral strain period reported that patients under the age of 50 exhibited worse outcomes from the total PLWHIV patient cohort [51]. This elevated risk was not reported in other sub-groups [51]. A second study, again from New York during the Ancestral strain period, demonstrated that HIV is a contributing factor directly that leads to a worse clinical prognosis, including ICU admission [56]. This poor prognosis is magnified among patients with an advanced stage of the disease [53,56]. Finally, meta-analyses conducted during the pre-Omicron period have so far generated conflicting results. Some report a greater mortality risk in PLWHIV, while others do not report any irregularities in outcomes between controls and patients with HIV [57,58,59,60,61].

Limited and heterogeneous findings exist regarding the relevance of antiretroviral treatment (ART) on the progression of COVID-19 in individuals infected with HIV. Some studies have suggested that patients on ART experienced milder COVID-19 symptoms and had a better prognosis compared to the general population during the pre-Delta period, leading to a quicker symptom resolution [43]. However, other studies during the Ancestral strain period have failed to find evidence supporting the protective effect of ART [39]. A study from Spain during the Ancestral strain period reported that a specific ART is not correlated to specific outcomes [62]. In contrast, data from a large multicenter cohort study in Madrid, involving over 75,000 participants from HIV clinics again during the Ancestral strain period, indicated that the likelihood of hospitalization varied based on the ART regimens used [63].

In a retrospective analysis of linked health data across all variants, patients with HIV were reported to have approximately twice the likelihood of hospitalization following an acute SARS-CoV-2 infection versus controls [64]. This risk was especially prominent among those who were either unvaccinated or inadequately vaccinated and persisted both prior to and during the Omicron wave of the pandemic [64]. It is worth noting that most of this increased risk seems to be attributed to differences in sociodemographic factors and underlying medical conditions [64]. These outcomes have since been replicated across all variant periods (Table 2).

The evidence for whether HIV independently contributes to adverse outcomes in COVID-19 remains inconclusive. One meta-analysis, which pooled data from six studies, reported that PLWHIV had a greater risk of hospitalization versus controls [65]. The combined odds ratio for hospitalization was found to be 1.49 (95% CIs: [1.01–2.21]), with individual odds ratios from the included studies ranging from 0.69 to 2.19 [65]. However, systematic reviews conducted by Mellor et al. and Barbera et al. during the pre-Delta period, which explored the relationship between HIV and the risk of hospitalization, presented conflicting findings [57,66]. Two studies included in these reviews reported an increased risk of hospitalization among individuals with HIV when compared to controls [53,67]. Conversely, other studies, during the Ancestral strain period, found no significant variation in the risk of hospitalization based on HIV status [42].

In conclusion, although various studies early in the pandemic reported mixed findings on HIV and COVID-19 risks, it is now the consensus that HIV is a risk factor that contributes to poor clinical outcomes irrespective of the variant. As with immunocompromised patients, it is anticipated that earlier variants exhibited an increased risk of mortality, especially due to the fact that immunization rates and healthcare services improved later in the pandemic.

### 3.3. CLD

CLD is widespread, affecting an estimated 112 million individuals across the world [68]. CLD often leads to hepatic decompensation and hepatocellular carcinoma (HCC), resulting in approximately 2 million annual deaths [68]. The presence of cirrhosis in CLD patients is associated with immune dysregulation, causing concern about potential complications for these patients when infected with the SARS-CoV-2 [68].

Data from a multinational study on CLD cohorts during the Ancestral strain period with confirmed SARS-CoV-2 infection focused on the association between baseline liver disease severity and outcomes. The findings indicate that the severity of liver disease at baseline plays a significant role in determining the outcome [68]. As CLD progresses from non-cirrhotic stages to different stages of cirrhosis based on the Child–Pugh classification, the risk of adverse outcomes such as the need for ICU admission and death increases [68]. Although patients without cirrhosis have similar outcomes to individuals without liver disease, those with cirrhosis face a significantly elevated risk (Table 3). In this study, patients with cirrhosis experienced a mortality rate of 32% [68].

Furthermore, this study demonstrated a progressive escalation in the risk of death for each stage of liver disease when compared to a control cohort of UK patients without CLD who tested positive for the SARS-CoV-2 virus during the Ancestral strain period [68]. These trends persisted even when the analysis was focused on individuals, with different cirrhosis stages showing a statistically significant elevated risk of death with increasing cirrhosis severity [68]. The co-occurrence of infection and cirrhosis presents a particularly severe threat, as it combines immune dysregulation, viral infection and impaired coagulation [68].

The National COVID Cohort Collaborative (N3C) completed a study which encompassed nearly 221,000 patients with CLD and identified a 2.38-fold (95% CIs: [2.18–2.59]) adjusted hazard ratio (aHR) for the increase in all-cause mortality risk in patients with cirrhosis following SARS-CoV-2 infection during the pre-Omicron period. Additionally, among all patients with cirrhosis CLD, the aHR of the likelihood of all-cause mortality within 30 days increased by 3.31 (95% CIs: [2.91–3.77]) [69]. These findings highlighted a lower overall incidence of 30-day mortality during the pre-Omicron period from all causes versus previous findings (8.9% vs. 17%) in cirrhosis cohorts [69]. These results are consistent with prior studies, which also demonstrated higher hospitalization rates in infection-free CLD groups [69]. The observed increase in hospitalization rates can likely be attributed to healthcare protocol modifications implemented during the pandemic, such as the adoption of standardized testing before hospital admissions [69]. Patients with cirrhosis aged under 50 years, had an aHR for 30-day mortality of 1.59 (95% CIs: [1.23–2.05]) [69]. This aHR further increased to 3.03 (95% CIs: [2.68–3.42]) for individuals above the age of 65 years [69]. The adjusted risk ratio (aRR) for vaccinated patients with respect to hepatic dysfunction was reported across the Delta and Omicron lineages [70]. Specifically, the aRR was 1.38 (95% Cls: [1.10–1.73]) for hepatic dysfunction patients when infected by the Delta variant, while the aRR was 1.23 (95% CIs: [1.05–1.44]) for the BA.1 variant, and the aRR was 1.51 (95% CIs: [1.36–1.68]) for post BA.1 [70].

Similarly to the immunocompromised and PLWHIV populations, patients with chronic liver diseases with cirrhosis exhibit worse clinical outcomes with earlier variants of the virus. Importantly, the hereogenous nature of CLD and underlying etiology that leads to this chronic disease contribute strongly to the variable clinical outcomes following a SARS-CoV-2 infection. Nevertheless, these studies summarize that liver functionality is directly linked to COVID-19 clinical outcomes.

### 3.4. Pediatric Patients

The impact of emerging virus variants on the severity of COVID-19 in children remains limited [72]. A dataset of 372,989 pediatric COVID-19 cases was analyzed in terms of clinical outcomes across all variants to determine the prevalence of different high-risk groups [72]. The findings revealed that pre-Omicron variants such as Alpha reported an increased risk of mortality or other poor clinical outcomes [72]. Equally, there was an elevated risk of in-hospital mortality associated with the Gamma variant relative to more recent variants that have subsequently emerged [72]. Interestingly, regardless of the variant, infants displayed the most severe prognosis. The most common variants observed among pediatric patients were the Omicron and Delta strains [72,73,74].

An increase in hospital admissions among pediatric patients was recorded during periods of high Delta and Omicron prevalence [75,76,77]. Regarding COVID-19 hospitalization criteria, these encompass various factors such as respiratory distress, complications arising from underlying comorbidities, and social determinants impacting healthcare accessibility [75]. Discrepancies in the reported rise in hospitalizations could potentially be attributed to the lack of vaccination among pediatric populations [75].

Despite the sudden surge in infection rates in children primarily driven by the emergence of new variants, our understanding of how these variants affect the pediatric airway remains limited. For instance, the Delta strain has been associated with increased odds of exposure to a wider pediatric population and, as a result, increased hospitalization rates [75,76,77]. However, a UK study investigated outcomes in the pediatric population during the periods in which the Alpha/Delta strains were mostly circulating, and revealed that these strains have similar disease duration, with the majority of symptoms resolving within 5 days [72,78]. Disease burden and duration was only marginally higher with Delta compared to other pre-Omicron variants [72,78]. This research therefore suggests that these strains result in similar disease patterns in terms of symptoms, which are characterized by a brief duration [78]. Finally, it has been demonstrated that infection by either the Alpha or Delta variants did not lead to worse clinical outcomes relative to other variants [79]. These findings imply that the increased hospitalization rates observed with the emergence of the Delta variant were not due to its heightened pathogenicity but rather a consequence of its increased prevalence [75,78,79].

According to Dowell et al., there is potential cross-reactivity between different variants of the virus [80]. The authors reported that children who have been exposed to the original strain of the virus developed a cross-reactive immunity to subsequent pre-Omicron variants [80]. Interestingly, the levels of these cross-reactive antibodies were found to be higher in children six months after infection compared to adults [75,80]. In the pediatric population, the duration of the disease is dependent on its severity [81]. It has been observed that the duration exceeds 10 days for all patients, while critically ill patients experience a duration of over 20 days [81,82,83,84]. In general, earlier strains, such as the Alpha and Gamma strains, lead to higher hospitalization rates for pediatric patients [72,79].

Various studies have reported varying lengths of hospital stays in pediatric patients. The minimum and maximum average stays recorded were approximately 8 and 15 days accordingly [82,83]. The mean in-hospital duration was found to be 14 days [81,82]. Qiu et al. concluded that a moderate disease burden results in a greater in-hospital duration versus milder symptoms [85]. Notably, individuals infected with the Delta variant appear to have a significantly longer median hospital stay of 5.7 days [86]. Generally, children admitted to the Pediatric Intensive Care Unit recover without any adverse outcomes [81]. However, fatal outcomes have been reported, particularly among patients with underlying health conditions [81,84]. Similarly to other special populations, immunization minimizes the impact of COVID-19 on clinical outcomes and duration of symptoms, although not as effectively in Omicron-strain infections [87]. Table 4 summarizes clinical outcomes in the pediatric population.

### 3.5. Limitations

The limitations of this scoping review include the lack of the use of a systematic quality assessment tool for the selected articles. Secondly, selection bias might have been introduced as articles were excluded during the filtering process based on titles and/or abstracts. Thirdly, the included articles have significant heterogeneity in terms of study design and included patient populations which may complicate the interpretation of clinical outcomes. Also, additional search strings might have been able to identify further articles that were relevant for this work. Furthermore, the evolving nature of the pandemic, emerging variants, population immunity and re-infections, and the availability of anti-viral therapeutics and vaccines might potentially skew the clinical outcomes and result in studies incorrectly reporting more clinically severe outcomes for earlier variants. While this review highlights the general impact of SARS-CoV-2 variants, the specific effects of various comorbidities within these special populations need further investigation. The long-term health outcomes of COVID-19 in special populations remain underexplored. Most studies focus on immediate clinical outcomes such as hospitalization and mortality rates. Further research is needed to investigate the long-term effects of COVID-19, particularly in immunocompromised individuals, PLWHIV, pediatric patients, and those with chronic liver disease.

## 4. Conclusions

Overall, it is vital to recognize the valuable data that are collected from the infection of different SARS-CoV-2 variants and their impact on clinical outcomes in the general population. For the purposes of this work, however, we focused on immunocompromised patients, PLWHIV, CLD patients, and pediatric patients. In summary, earlier SARS-CoV-2 variants appear to have resulted in poorer clinical outcomes across these special populations. Nevertheless, patient heterogeneity, study design variation, and immunization differences among pandemic epochs all contributed to a more convoluted clinical image for these special populations. Other factors, such as the use of immunosuppressive drugs or the presence of cirrhosis in CLD patients are clinical characteristics that reinforce poor clinical outcomes following a SARS-CoV-2 infection. Nevertheless, it would appear that recently circulating SARS-CoV-2 variants remain relatively weaker compared to the ancestral lineages in terms of the severity of clinical outcomes in these special population patients. Furthermore, this investigation has demonstrated the variability in the clinical severity experienced by various special populations following an infection from different SARS-CoV-2 variants. The SARS-CoV-2 pandemic has, therefore, emphasized the crucial role healthcare professionals and registries hold in documenting such clinical outcomes in different patient sub-populations relative to the evolution of different variants of the virus.

## Figures and Tables

**Table 1:** Selected studies on immunocompromised patients.

Included Studies	Type of Study	Population Size (N)	Type of Variant	Clinical Outcomes (% of Patients) *
Singson J.R.C. et al., 2022 (USA) [35]	Observational Study	N = 22,345	54.2% pre-Delta, 20.0% Delta, 25.8% Omicron for immunocompromised	ICU admission (OR): 1.26; 95% CIs: [1.08–1.49] Mortality (OR): 1.34; 95% CIs: [1.05–1.70]
Yadaw A.S. et al., 2023 (USA) [38]	Retrospective Cohort Study	N = 2,453,799	Ancestral, Alpha, Beta, Gamma, Delta, and Omicron	Hospitalization: 16.1% (AID), 16.5% (IS) Life-threatening: 4.5%(AID), 4.9% (IS)
Esper F.P. et al., 2023 (USA) [44]	Retrospective Cohort Study	N = 2779	48.8% Alpha, 5.2% Gamma, 34.2% Delta, 25% Omicron Any immune co-morbidity: 15.7% Alpha, 13.9% Gamma, 19.4% Delta, 14.3% Omicron	ICU admission: 2.7% (Alpha), 3.3% (Gamma), 3.6% (Delta), 1.0% (Omicron) Mortality: 1.3% (Alpha), 1.6% (Gamma), 1.0% (Delta), 0,4% (Omicron)
de Prost N. et al., 2022 (France) [45]	Observational Study	N = 259	N = 111 Delta (86.4% immunosuppressed), N = 148 Omicron (56.8% immunosuppressed)	Invasive MV: 56.8% (Delta), 46.6% (Omicron), 51.6% (Omicron, only immunosuppressed) No differences among Omicron sub-lineages on 28-day mortality
Evans R.A. et al., 2023 (UK) [46]	Observational Study	N = 11,990,730	Omicron	ICU Admission: 16.5% Mortality: 7.4%
Ketkar A. et al., 2023 (USA) [47]	Retrospective Cohort Study	N = 16,873,161	2.7% immunocompromised Alpha, Beta, Gamma, Delta, and Omicron	Hospitalization: 23.5%
Malahe S.R.K. et al., 2023 (Netherlands) [48]	Prospective Observational Study	N = 114	Omicron	Hospitalization: 20% Mortality: 1 patient
Huygens S. et al., 2024 (Netherlands) [49]	Prospective Cohort Study	N = 245	50% Omicron, 50% Delta	MV or Mortality: 27%
Turtle L. et al., 2023 (UK) [50]	Prospective Cohort Study	N = 156,552	14% immunocompromised Ancestral, Alpha, Delta, and Omicron	Ancestral Strain Mortality (OR): 1.28; 95% CIs: [1.20–1.36] Alpha Strain Mortality (OR): 1.19; 95% CIs: [1.12–1.26] Delta Strain Mortality (OR): 0.83; 95% CIs: [0.76–0.91] Omicron Strain Mortality (OR): 0.66; 95% CIs: [0.54–0.80],

MV: mechanical ventilation; AID: autoimmune disease; IS: immunosuppressant; OR: odds ratio; CI: confidence intervals. * Percentage of patients included in this column unless otherwise stated (e.g., OR).

**Table 2:** Selected studies on PLWHIV.

Included Studies	Type of Study	Population Size (N)	Type of Variant	Clinical Outcomes (% of Patients) *
Yang X. et al., 2021 (USA) [40]	Retrospective Cohort Study	N = 1,436,622	Ancestral, Alpha, Gamma	Hospitalization (OR): 1.20; 95% CIs: [1.15–1.26] Mortality (OR): 1.29; 95% CIs: [1.16–1.44]
Bertagnolio S. et al., 2022 (Switzerland) [43]	Prospective Cohort Study	N = 197,479	Ancestral, Alpha, Gamma	Hospitalization: 38.4% Mortality: 24.3%
Bhaskaran K. et al., 2021 (UK) [55]	Prospective Cohort Study	N = 17,282,905	Ancestral	Mortality (HR): 2.90; 95% CIs: [1.96–4.30]
Tesoriero J.M. et al., 2021 (USA) [56]	Retrospective Cohort Study	N = 2988	Ancestral	Hospitalization (sRR): 1.38; 95% Cls: [1.29–1.47] Mortality (sRR): 1.23; 95% Cls: [1.07–1.40]
Del Amo J. et al., 2020 (Spain) [63]	Retrospective Cohort Study	N = 77,590	Ancestral	Hospitalization (SR): 17.8; 95% CIs: [17.7–18.0] Mortality (SR): 3.7; 95% CIs: [3.6–3.8]
Puyat J.H. et al., 2023 (Canada) [64]	Retrospective Cohort Study	N = 253,129	Alpha, Gamma, Delta, Omicron	Hospitalization: 17.5% Mortality: 1.2%

sRR: standardized rate ratio; SR: standardized risk; HR: hazard ratio; CI: confidence intervals. * Percentage of patients included in this column unless otherwise stated (e.g., HR, SRR).

**Table 3:** Selected studies on patients with CLD.

Included Studies	Type of Study	Population Size (N)	Type of Variant	Clinical Outcomes (% of Patients)
Marjot T. et al., 2020 (UK) [68]	Retrospective Cohort Study	N = 745	Ancestral	Mortality: 8% without cirrhosis Mortality: 32% with cirrhosis Mortality: 19% with Child-Pugh class A Mortality: 35% Child-Pugh class B Mortality: 51% Child-Pugh class C
Ge J. et al., 2021 (USA) [69]	Retrospective Cohort Study	N = 220,727	Ancestral, Alpha, Gamma, Delta	Hospitalization by day 90: 22.9% (Non-cirrhosis) Hospitalization by day 90: 50.1% (Cirrhosis) Mortality by day 90: 2.3% (Non-cirrhosis) Mortality by day 90: 12.7% (Cirrhosis)
Griggs E.P. et al., 2024 (USA) [70]	Retrospective Cohort Study	N = 60,488	Delta, Omicron	Hospitalization (Delta): 6.6% Hospitalization (BA.1): 8.1% Hospitalization (BA.2): 7.2% Hospitalization (BA.4/BA.5): 7.5% Hospitalization (Post-BA.4/BA.5): 6.8%
Velásquez Garcia H.A.V.et al., 2024 (Canada) [71]	Retrospective Cohort Study	N = 162,509	11.2% Alpha, 8.6% Gamma, 25.7% Delta, 4.3% Omicron	Hospitalization (No Cirrhosis): 3.6% Hospitalization (Cirrhosis): 17.2% ICU admission (No Cirrhosis): 1.5% ICU admission (Cirrhosis): 12.8%

**Table 4:** Selected studies on pediatric patients.

Included Studies	Type of Study	Population Size (N)	Type of Variant	Clinical Outcomes (% of Patients) *
Maldonado-Cabrera A. et al., 2023 (Mexico) [72]	Retrospective Cohort Study	N = 372,989	Alpha, Gamma, Delta, Omicron	Hospitalization (IRR): 1.69 (Alpha); 95% CIs: [1.54–1.86], 2.67 (Gamma); 95% CIs: [2.52–2.82], 0.97 (Delta); 95% CIs: [0.96–0.98], 0.95 (Omicron); 95%: [0.94–0.96] Mortality (IRR): 1.68 (Alpha); 95% CIs: [1.52–1.85], 2.7 (Gamma); 95% CIs: [2.54–2.85], 0.99 (Delta); 95% CIs: [0.98–1.00], 0.97 (Omicron); 95% CIs: [0.96–0.98]
Edward P.R. et al., 2022 (USA) [79]	Retrospective Cohort Study]	N = 714	13.4% Alpha, 5.3% Gamma, 16.7% Delta, 30.1% Omicron	Hospitalization (OR): 2.2 (Alpha); 95% CIs: [0.73–6.6], 6.7 (Gamma); 95% CIs: [2.0–22.1], 2.8 (Delta); 95% CIs: [0.95–8.0], 0.79 (Omicron); 95% CIs: [0.20–2.7] ICU Admission (OR): 2.5 (Alpha); 95% CIs: [0.53–13.6], 5.4 (Gamma); 95% CIs: [0.92–31.4], 2.7 (Delta); 95% CIs: [0.56–14.8] 4.5 (Omicron); 95% CIs: [0.94–24.1]
Martin B. et al., 2022 (USA) [88]	Prospective Cohort Study	N = 167,262	Delta, Pre-Delta	IMV: 6% (Delta) IMV: 8% (Pre-Delta)
Butt A.A. et al., 2022 (Qatar) [89]	Retrospective Cohort Study	N = 1735	Delta, Omicron	Moderate Disease: 15.7% (Delta) Moderate Disease: 2.2% (Omicron)

IRR: Incidence Rate Ratio; OR: Odds Ratio; IMV: Invasive Mechanical Ventilation. * Percentage of patients included in this column unless otherwise stated (e.g., OR, IRR).

## Data Availability

No new data were created or analyzed in this study. Data sharing is not applicable to this article.
